# Letter from Dr. Beaugrand

**Published:** 1839-10-12

**Authors:** 


### FOREIGN CORRESPONDENCE.

### LETTER FROM DR. BEAUGRAND.—No. IV.

*On the Radical Cure of Hernia—M. Gerdy's Plan.*

To the Editors of the Medical Examiner.

PARIS, 15*th July*, 1839.

FROM the earliest times, the radical cure of hernia has been an object of medical attention. The ill success of the various means employed to effect this purpose had at length decided the surgeons of the last century to abandon it, until lately the labours of MM. Belmas and Jameson again awakened the notice of surgeons and the hopes of the afflicted.

In 1834, M. Gerdy conceived an entirely new project, which he put in practice, for the first time, in 1835, and which he has, since, a considerable number of times repeated with nearly unvarying success. Attentive observation of the results of the operation has enabled him to apply some important modifications to the manual process for the relief of this affection. Several patients having been recently submitted to the operation at the hospital La Charité, M. Gerdy has had an opportunity of showing to his pupils his mode of practice, and the results which attend it. We propose to offer your readers a recapitulation of these lectures, which are of special practical interest, and to preface them with some remarks upon the methods anciently or more recently proposed for the radical cure of herniæ.

The first fact which strikes us in looking over the list of authors who have treated of herniæ, is, that the old writers devoted themselves almost exclusively to the radical cure of these lesions, whilst those of later times have given more particular attention to that very alarming complication, strangulation. Has this been owing to the disgust occasioned by repeated failure, or is it but one of those singular caprices of which history furnishes so many instances, which have at times consigned to oblivion many useful inventions, afterwards to revive them, with all the honours of novelty? Up to the time of Franco and of Ambrose Paré, it was not proposed to operate for strangulated hernia. Celsius is opposed to any operation in this case, (Book vii., chap. 20.) Astius pushes his timidity so far as to delay attempts at reduction so long as the symptoms do not moderate, when he recommends the scarification of the inflamed parts. Such were the precepts which influenced surgeons until the sixteenth century. Let us examine the plans adopted for the cure of hernia. They may be divided, according to Guy de Chauliac, into two classes. The first comprehends external treatment, topical applications, compresses, rest, and regimen; the second, manual operations.

It is incontestable, that the employment of the means embraced in the first class is capable of effecting a radical cure. No further result can, however, be obtained, than the closing of the ring; and, even if the obliteration of the neck of the sac took place, the parts would be merely restored to the condition in which they were previous to the formation of the hernia. Under the influence of a fresh exciting cause, the protuberance of the intestine must again take place, there being no barrier to arrest its progress. To this first objection to the methods in question may be added others. First, their uncertainty: of the multitude of persons who wear trusses, it is well known that there are very few who are completely cured. Nor is absolute rest a more certain means. A few rare cases are cited, and cited because they are rare, of cures of hernia effected during prolonged rest in various diseases; but how many individuals are almost daily met with, who, after an absolute confinement of months, as in fractures, for example, retain the ruptures with which they had been previously affected. As for the employment of astringents, admitting their occasional efficacy, the great length of time which they require to effect a cure, according to the reports of their advocates, is a sufficient objection. M. Beaumont, in a work on the subject, (Paris, 1827,) reports out of fifteen cases of inguinal hernia, fourteen cured after a mean period of two years.

The second class comprises surgical operations. From remote antiquity, these have been resorted to for the cure of hernia; they may be reduced to four heads. 1st, it was attempted to produce inflammation, and even the destruction of the hernial opening, by means of caustics, with a view to subsequent cicatrization; 2dly, the obliteration of this aperture was sought by means of ligatures employed in various manners; it was, by others, thought that the incision of the integuments and of the sac, and even their complete excision, would bring about a suppuration of the parts likely to result in the formation of a solid cicatrix, which would obstruct the passage of the intestines; 4thly, others, finally, have proposed to block up the ring, by introducing a substance capable of adhering to it,—of this nature is the plan of M. Gerdy. These various methods have often been combined one with the other; thus, certain surgeons of the middle ages used the caustic, or a ligature, after having made an incision; but, in these cases, the cauterization or the ligature was the essential part of the operation.

I shall not dwell upon the dangers of cauterization. It is sufficient to read the works of those who have advocated this plan, as Brunus, Lanfranc, Guy de Chauliac, &c., to see that it was never employed without anxiety, and that it was followed by formidable, and even mortal consequences. Lanfranc, particularly, inveighs against the detestable eagerness of surgeons who hesitate not to put a little gold in the balance with the lives of their patients, and expose their victims to violent measures, his own proceeding being, however, of course, excepted. Whatever success may then be anticipated from this method, it has been for ever banished from science. The idea of closing the hernial aperture with a ligature, to procure its obliteration, nearly in the manner that wounds are closed with a suture, very naturally suggested itself to the mind of surgeons. Thus, we see this practice mentioned in the oldest times, (see Actius, Paul of Egina,) and receive considerable extension in the middle ages, (see Brunus, Theodoric, Roger of Parma, &c.,) at the same time that it was modified in its application. There were two principal modes employed, according as the spermatic cord was or was not implicated, and, afterwards, according as the testicle was or was not involved. I shall not enter into the details of these various operations; I shall merely observe, that, in our own times, M. Bonnet, of Lyons, has proposed a kind of suture, of the contrivance and results of which he has published an account in the Journal des Connaissances Medico-Chirurgicales, for July, 1836. "This methed consists in inserting pins, near the ring, through the hernial envelopes, and so arranging them as to keep in contact the walls of the sac, leaving them in place until the development of adhesive inflammation." At the time mentioned, the author could bring to the support of his plan but four successful cases,—he has since obtained a larger number. Does this plan meet the end in view for the radical cure of hernia? Does M. Bonnet suppose that it is always very easy to avoid wounding the spermatic vessels, particularly when they have been somewhat scattered by the hernial tumour? And, if the sac has been reduced, what will be effected by a suture passing merely through the skin? Besides, even if the sac remain without the ring, as generally happens, is it very easy to pinch it up with the skin, to insert the pins through it?

As for the methods of incision, excision, castration, &c., no danger is to be apprehended of an attempt to revive them: we shall merely observe that incision, *without opening the sac*, may be employed in certain cases in which invagination is impracticable, as will be seen further on.

The last method which can be classed under the head of surgical operations is that of the *obliteration* of the hernial canal. In this case, a body, organized or not, is introduced into the canal, in order, by its presence, to develop an inflammation, which, involving the surrounding parts, may stop up the canal. Such is the plan of plugging up the ring with the testicle, described and highly censured by St. Moinichez and by Seultat. Such is the method of M. Belmas, who introduces into the neck of the sac a little bladder of oxen's gut, or filament of the same covered with the gelatine of bird-nests. Such is the insertion (pelotonnement) of the hernial sac in the ring after the operation for strangulated hernia, proposed by Garengeot, (Surgical Operations, ed. of 1718,) and condemned by Louis, (Memoir of the Roy. Acad. of Surg., vol. xl. p. 471, ed. in 12mo.) Such, in fine, is the well known operation of M. Jamelon.

Struck with the inconveniences and dangers of these different methods, and satisfied of their inefficacy, M. Gerdy, as we set out with mentioning, suggested in 1834, and practised in 1835, a new mode of obliteration, which had never previously been attempted. This process, called by the author invagination, consists in obliterating the hernial canal by means of a wedge formed at the expense of the skin of the scrotum. This skin, pushed by the fingers into the canal, is there retained by a few threads of a suture. At first M. Gerdy employed five threads, afterwards but three, and, for a long time, he has been satisfied that two, or even a single thread, sufficed to effect the most prompt and solid adherence of the invaginated skin. The inflammation of the latter was immediately brought about by means of a piece of charpie or cotton impregnated with ammonia. Finally, in the first operations, the groin was covered with a bladder of cold water, and the threads were removed the fifth day.

Let us follow the details of this operation. For a few days previously, prudence requires dieting on the part of the patient; the evening before the operation he is to be purged with

Seidlitz powders, and on the morning of the day he is to have an emollient injection. The object of these precautions is to obviate the necessity of the patient's going to stool during some days immediately following the invagination.

The instruments necessary for the operation are, first, a curved needle, flattened, and sharp at both edges, eight or ten inches long, and about a line and a half broad—with the eye about four lines from the pointed extremity, the other extremity terminating in a flat button, by which the needle is moved backwards and forwards in the porte-needle. This has been expressly contrived by M. Gerdy, and consists of a steel blade, three lines in diameter and five or six inches long, set in a wooden handle, with a curve at its extremity similar to that in the needle; the entire concavity of this instrument has a groove for the needle to slide in, with the edges turned in, in order to retain the latter in place; secondly, a number of rolls of adhesive plaster, or of gum elastic sounds, seven or eight lines in length, and three in diameter; and, thirdly, some coarse waxed thread. In using the instrument, a double thread is passed through the eye of the needle, the extremity of which is tied and hangs out of the eye only about an inch, that it may be the more readily withdrawn. During the operation, the thread accommodates itself very well to the groove of the porte-needle, along the concave face of the needle.

Every thing being properly disposed, the scrotum and inguinal region having been previously shaved, and the hernia perfectly reduced, the patient is placed upon his back, his head supported with pillows to relax the muscles of the abdomen, the legs separated and supported by assistants, in a word, nearly in the position for the sub-pubic operation of lithotomy. The surgeon places himself between the legs of the patient, and, resting the extremity of his left index-finger upon the lateral parts of the scrotum, about an inch from the root of the penis, presses upon the skin, pushes it up in the direction of the spermatic cord, enters the inguinal ring, and thus thrusts the integuments into the very bottom of the canal. The skin is thus made to form an actual invagination, in the form of the finger of a glove, which fills up the hernial canal, and shuts it up like a stopple. To keep this stopple in place is now attempted by means of a suture. For this purpose, keeping his index-finger in the situation described, the operator slips along the palmar side of his finger, which looks forward, the extremity of the convex side of the porte-needle, the handle of which he holds with his right hand nearly perpendicular to the axis of the body, and thus introduces it to the bottom of the cul-de-sac. The extremity of the porte-needle is thus placed in front of the finger, which even rests upon the instrument and prevents its approaching the belly, while the needle is rapidly thrust along and carried through the most distant portion of the invaginated skin, the whole anterior paries of the inguinal canal, and the skin which covers it. The point of the needle is thus brought out externally; it is sometimes a little difficult to effect this, as the integuments of the groin yield before the point of the instrument, and are pushed forward rather than penetrated. It will be accomplished with greater ease, by depressing the integuments on each side of the joint, either with the finger of an assistant, or dissecting forceps. When the needle has emerged beyond the level of the eye, the thread is disengaged, the tied extremity remaining without, and the instrument is drawn back into the canula, which had remained at the bottom of the cul-de-sac. The porte-needle is withdrawn, the needle is again threaded with the extremity of the double thread which has passed through the cul-de-sac—the left index-finger still occupying the same place. The instrument is then introduced a second time, by means of the same manœuvre, care being taken to pierce the integuments about two or three lines outside of the first wound. The thread being disengaged from the eye, and the needle withdrawn, the invaginated cul-de-sac is retained at the bottom of the inguinal canal, that is to say, an inch and some lines above the ring, by means of the double thread, the ends of which remain without. If the canal is very large, a second thread may be placed in the same manner, outside the first. That being done, the next object is to fasten the extremities of the suture, which is done as for the ordinary quill suture. One of the rolls of which we have spoken is passed through the tied extremity, and by pulling the other end, the cylinder is brought in contact with the skin; a second roll is placed between the two ends forming the other extremity, and they are twisted in such a manner as to allow the suture to be loosened in case of necessity. It will be seen that the invaginated cul-de-sac is fixed in situ by a ligature, the middle of which passes through its bottom, while the extremities are fastened externally upon the rolls—the strangulation of the soft parts included, which occurs in the use of other sutures, being thus avoided.

The operation being terminated, the patient is kept in bed, with a pillow under his hips, and a cushion under the scrotum, to keep that organ elevated and in apposition with the pubis. The weight of the testicles is thus prevented from interfering with the perfect rest of the inguinal regions. Upon the latter are applied compresses, soaked in tepid or cool water, according to the season, and the patient is subjected to diet for two or three days.

What takes place during this operation? Sometimes, in pushing up the skin, the peritoneal or hernial sac is also pushed up: in this case, it is behind the finger, and the needle does not implicate it. The same may be said of the epigastric artery. For these parts to be wounded, the instrument, instead of penetrating from behind forward, must get to the other side of the finger of the operator in search of the organs protected by the finger, which is quite impossible. This is established as well by reasoning based upon an anatomical knowledge of the parts, as by the numerous cases upon which the operation has been performed. It may happen that the hernial sac is adherent, in which case it will accompany the skin pushed up, and the invagination will take place in its interior; but, generally, the skin of the scrotum, being loose and moveable, slips in front of the sac, and places itself between it and the ring. The pain during the operation is ordinarily slight; the puncture is, however, tolerably painful; and those who analyze their sensations feel distinctly the double wound which the needle makes, first in passing through the skin of the cul-de-sac, and afterwards through that around the outside of the inguinal region.

After the operation, there follows a very remarkable series of phenomena, of which the surgeon should be apprised. In the first place, the presence of the thread and of the portion of invaginated skin, determines an inflammation of the cellular tissue enclosed in the inguinal canal. This inflammation, which supervenes in a few moments, is perfectly established at the end of twenty-four hours, and the adhesion between the plug and the walls of the canal is at this period sufficiently formed for the thread to be removed without inconvenience. If the parts are then touched, there are felt not only the invaginated cylinder, but a hard kernel, slightly sensible to pressure, which is about an inch long.

The following day, the adhesion becomes more and more intimate, and the kernel presents still more hardness. The threads are then to be withdrawn: M. Gerdy usually takes them out the third day, often the second, and, in one case, to which we shall allude further on, they were withdrawn at the expiration of thirty hours. To get the threads out, the end of the twisted thread is cut under the cylinder of adhesive plaster, or of gum elastic sound; the other cylinder is then withdrawn with the loop which kept it fixed. From the openings through which the points of the suture passed, there escapes pus, sometimes in considerable quantity. This pus must be daily, and even several times a day, evacuated, by slightly compressing the surrounding tissues; and as these small openings are easily obliterated by the pus itself, which coagulates and forms scabs at their orifices, at every dressing the scabs should be removed with the point of a stylet. The suppuration generally dries up at the end of seven or eight days; the limits of the inflammatory kernel are then much contracted; a hard cylinder is felt in the inguinal canal, which closes it exactly, and resists the weight of the intestines, when the patient exerts himself. At a later period, the invaginated cul-de-sac is transformed into a fibrous cord, which may be readily perceived for some time in persons who have been operated upon. The same thing happens here as in the case of arteries, the calibres of which have been obliterated,—that is to say, the cutaneous plug is gradually absorbed, and, after a lapse of time, disappears altogether, leaving the canal in its primitive healthy condition, as it was before the occurrence of the hernia. As for the hernial sac, the portion contained in the canal undergoes adhesive inflammation, and is soon completely obliterated. This fact we ascertained two years since, in a subject who died of sporadic cholera, the eighth day after the operation, without the supervention of the slightest local accident.

While these phenomena have been taking place, a purulent secretion is observed in the cul-de-sac, proceeding from the part through which the threads had passed. Still later, the invaginated portion ulcerates at the level of the external orifice, separates from the skin of the scrotum, and the fold formed by the integuments at the level of the ring descends. By degrees, the lips of this opening close, and are finally so completely effaced by the retraction of the surrounding skin, that, at the end of twenty or thirty days, there is barely visible a small cicatrix, which is lost in the rugæ and hair of the scrotum.

What are the accidents which may complicate this trifling operation? In the first place, the inflammation necessary to procure the union of the parts may exceed ordinary limits, and spread to the cellular tissue of the inguinal region. The free application of leeches, venesection, if there be febrile reaction, the prolonged use of the warm bath, with particular attention to keeping the patient quiet, and, finally, starch poultices, will not fail to arrest the progress of the inflammation, and bring it to a favorable termination. An attack of erysipelas should be combated by the same remedies; and, if there should be any considerable tumefaction of the parts, it would be advisable, in case the threads have yet not been withdrawn, to take them out at once, to avoid the strangulation and gangrene of the parts enclosed. The accident most to be apprehended, *a priori*, would be peritonitis; yet, what is not a little remarkable, this symptom did not once show itself, in more than fifty patients subjected to the operation. It is not, therefore, worth while to dwell upon it.

Is the operation under notice applicable to all cases of hernia, or is it liable to the reproach addressed to lithotrity, that its subjects must be selected? But, except in the cases in which the patient is otherwise doomed to certain death, may not the same be said of all surgical operations? are they not all liable to counter-indications? and why should the means of relieving a certain number of sufferers be rejected, because the relief cannot be extended to all? We do not hesitate frankly to admit, that the subjects must be selected for the operation. But, the motives for exclusion are few in number, and may hereafter be diminished. Thus, formerly, M. Gerdy, not willing to risk the success of his operation, did not care to apply it to cases of congenital hernia, till, in the course of the year 1836, he operated upon a lad, fourteen years of age, affected with a congenital hernia, which, from inaccurate information, was supposed to have been acquired. Not the slightest unfavourable symptom occurred; the threads were withdrawn at the end of thirty hours, and the cure was as rapid as satisfactory. There are subjects, afflicted with old herniæ, in whom the canal has acquired so great a volume, that only a part of it can be obliterated by an invagination. In such cases, M. Gerdy performs a double operation, introducing successively, at different times, the skin of the scrotum into the vast opening. He waits for the first operation to succeed before attempting a second, as the former may suffice to bring about the necessary closing of the ring. When, on the other hand, the latter is originally so narrow that the finger cannot be introduced, it is to be dilated by the repeated introduction of the little finger, when the operation is performed as under ordinary circumstances.

In women, the inguinal ring is excessively contracted, and an invagination of the skin almost impossible. Two years ago, M. Gerdy attempted the operation upon a young woman, but the integuments could not be properly pushed up; nothing more could be obtained than a small fold, a few lines in depth. The ordinary procedure had in this case to be laid aside; and the ring having been exposed by means of an incision of an inch in extent, made with the greatest precaution, a small tent of charpie was placed in it, which, being daily renewed, produced an inflammation, which resulted in the perfect obliteration of the inguinal ring. This woman has been recently seen by M. Gerdy, and at the time the cure remained perfect.

There are, therefore, no absolute or necessary reasons to exclude any case from the benefits of the operation, except the irreducibility of the hernia, and the bad constitution of the subject, which latter is a circumstance of prime importance in every surgical operation, however safe its character.

